# Examining HIV Risk and Exchange Sex Among Current and Formerly Homeless Young Adults

**DOI:** 10.1007/s10461-021-03364-z

**Published:** 2021-07-24

**Authors:** Danielle R. Madden, Sara Semborski, Eldin Dzubur, Brian Redline, Harmony Rhoades, Benjamin F. Henwood

**Affiliations:** Suzanne Dworak-Peck School of Social Work, University of Southern California, 669 West 34th Street, Los Angeles, CA 90089 USA

**Keywords:** Homelessness, Young adults, Exchange sex, Daily diaries, HIV

## Abstract

This study investigated HIV risk among homeless and formerly homeless young adults by examining risky sex behaviors (e.g., condomless sex, exchange sex, and sex with multiple persons) using 90-day and daily recall methods. Data came from a sample of young adults (aged 18–27) with current (*n* = 101) or past (*n* = 109) homelessness experience in Los Angeles, California, recruited between 2017 and 2019. Baseline surveys queried demographics and sexual history. Daily retrospective surveys queried sexual events. Multiple logistic regressions were used to test the effects of demographic characteristics including homelessness history, relationship status, substance use, and sexual history on risky sex outcomes. In this sample, 26% reported never using a condom during anal or vaginal sex in the past 90 days, 5% reported testing positive for HIV, 82% had limited to no knowledge of preexposure prophylaxis, and 8% reported having had exchange sex during a 7-day measurement period, with those experiencing homelessness more likely to report. The study suggests supportive housing can reduce the occurrence of exchange sex but that HIV prevention services are still needed in homeless and housing programs to promote safe sexual practices.

## Introduction

Homelessness is increasingly common among young adults aged 18 to 25, with 12-month estimates indicating a population prevalence in the United States of 9.5% [1]. Research has shown that these young adults who have experienced homelessness (YAEH) often face multiple challenges in addition to their immediate need for stable housing. YAEH have a disproportionate burden of HIV infection [2] compared to housed young adults, with estimates as high as 8% HIV seropositivity among YAEH [3–5]. Trauma is commonly experienced both prior to and after homelessness, such as emotional, physical, and sexual abuse [6]; institutional involvement through the foster care [6–11] or juvenile justice [6, 12] systems; and violence and victimization [13–15]. These experiences have been linked to increased risk behavior, including substance use [16–18] and risky sex [19, 20]. Elevated rates of substance use in particular have been connected to increased sexual risk behavior among YAEH [20, 21], who generally report low to no knowledge of preexposure prophylaxis (PreP) for HIV prevention [22]. Further underscoring the risk of HIV among YAEH are high rates of exchange sex, with estimates ranging from 11 to 41% and findings that the likelihood of participating in exchange sex increases with each additional year that a young person spends homeless [19, 23]. Research has identified that although some YAEH report engaging in exchange sex because of a general economic need, a smaller subset report engaging in sex specifically for access to housing [24].

To meet the needs of YAEH, homelessness service systems have increasingly implemented housing interventions with built-in supports, commonly referred to as supportive housing. Research has shown that supportive housing is effective at ending chronic homelessness for a target population that has an average age approaching 60 years old, yet it is unclear whether supportive housing effectively reduces sexual risk behaviors [25, 26]. In a 1-year longitudinal study of 421 chronically homeless adults aged 40 or older who transitioned from homelessness to supportive housing [25], sexual risk behavior changed over time, including an overall increase in the rate of sexual activity, but a decrease in rates of some sexual risk behaviors, including condomless sex and sex with multiple partners. Whether these patterns of sex risk behavior are similar for YAEH who are placed in supportive housing has not been investigated.

A limited number of studies have examined whether supportive housing programs adequately serve YAEH. Most studies relying on qualitative methods have found that YAEH feel that they have been positively affected by their participation in supportive housing programs [27–29]. At least one randomized controlled trial found that YAEH assigned to supportive housing reported better health and lower rates of substance abuse than controls without stable housing [30], as well as improvements in overall quality of life and leisure 2 years after move-in [31]. In a different study of HIV-positive YAEH, supportive housing was associated with a decreased viral load and increased CD4 count [32]. This supports the notion that HIV may be a “housing-sensitive condition,” which refers to health conditions whose transmissibility, course, and medical management are influenced by the presence or absence of stable housing [33].

The current study sought to build on this emerging literature by examining HIV risk and exchange sex among young adults who are either currently or formerly homeless after having been placed in supportive housing. We note that much of the existing literature that has examined YAEH has relied heavily on 30- to 90-day retrospective recall to capture sexual risk behavior. Although retrospective self-report data are easy to collect, their validity is often weak, referred to as reconstruction bias [34]. Reconstruction bias is a particular issue when the goal is to understand how experiences, behaviors, and events play out over time in specific contexts, requiring data with temporal resolution appropriate to the dynamics of the behavior of interest [35]. To address this methodological weakness, this study leveraged the extensive use of cell phones in this population [36] to capture daily reports of sex and sexual risk-taking behavior (i.e., any sex, condomless sex, sex with multiple partners, or exchange sex) during a 1-week period, which given this high-risk population was thought to provide ample time to examine risk behavior. Although evidence indicates that some forms of retrospective self-report are adequate for capturing sex events [37–39], it also suggests that longer-term retrospective methods become less valid as the nature of the risk increases, which is often accompanied by increased stigma [21]—e.g., substance use occurs in conjunction with sex [38], sex is condomless [39], and increased number of sexual partners [40].

The present study, which used daily recall in addition to 90-day recall of sexual risk behaviors among YAEH who are either currently or formerly homeless and now living in supportive housing, aimed to (a) describe predictors of risky sex during a weeklong observation period, (b) examine the role of housing status in daily sex risk behavior, and (c) determine if intensive longitudinal study designs, such as daily recall, can adequately capture risky sex behaviors in what is known to be a high-risk population. Although a main objective of this study was to advance methodological innovation to examine HIV-associated social determinants, we hypothesized that YAEH living in supportive housing will engage in less risky sex including exchange sex due to having their basic needs more met compared to those who are currently homeless.

## Methods

### Participants

Participants (*n* = 121 housed, 109 homeless) were recruited through flyers and informational sessions held at housing programs or drop-in facilities that served YAEH. Individuals were first asked to complete a self-administered screener on an electronic tablet that indicated whether they met the study’s eligibility criteria. Individuals were deemed eligible to participate if they could be interviewed in English, could read and understand smartphone items in English without assistance, and were willing to provide written informed consent. To be included in the housed sample, young adults had to have moved into a housing program between the ages of 18 and 25 years old and currently be no older than 27 years old. To be included in the unhoused sample, individuals needed to be between 18 and 25 years old and meet the McKinney-Vento Homeless Assistance Act [41] definition of homelessness that specifies lack of a fixed, regular, and adequate nighttime residence.

Study participants then completed a baseline questionnaire that consisted of items querying constructs such as demographics, HIV status, knowledge of PrEP, 90-day sexual history, and housing environment. Participants were then asked to complete a 7-day ecological momentary assessment (EMA) protocol that included a daily retrospective survey (i.e., daily diary) assessing any sex events that may have occurred during the previous day. A 7-day study period was selected because it was thought to be the minimal amount of time needed to capture adequate sex risk behaviors in a high-risk population using a high burden (i.e., daily) methodology. Through the use of an app developed for the study, participants could self-initiate and complete a previous day diary or would be prompted to do so at three points each day (e.g., 9 a.m., 12 p.m., 2 p.m.) that they select at the beginning of the week until that day’s diary was completed. The full protocol for this study is available elsewhere [42]. All protocols were approved by the Institutional Review Board at the University of Southern California.

### Measures

Demographics queried at baseline included age, gender (0 = *female or other gender* versus 1 = *male only*), sexual identity (0 = *other sexual identity*—i.e., gay or lesbian, bisexual, questioning or unsure, asexual, another orientation not listed versus 1 = *heterosexual*), ethnicity (0 = *non-Hispanic* versus 1 = *Hispanic*), race (0 = *other race*—i.e., American Indian or Alaska Native, Asian, White, Native Hawaiian or other Pacific Islander, South Asian, or other race not specified versus 1 = *Black*), and length of time without stable housing during lifetime (0 = *less than 1 year* versus 1 = *1 year or more*) [43]. Degree of problematic substance use was assessed with the 4-item CAGE Substance Abuse Screening Tool, which indicated if a respondent is consuming substances in a pattern (alcohol and other drug use) that is indicative of abuse problems (1 = *problem use*, 0 = *nonproblematic use*) [44]. Response options were reduced due to distributions in the sample (e.g., sexual identity, race); furthermore, a 1-year cutoff was chosen as the best approximation of chronic homelessness, which is defined as “individuals or families that have either been continuously homeless for 1 year or more, or have had at least four episodes of homelessness in the past 3 years,” per the National Academies of Sciences [33].

To better understand past and recent sexual behavior, participants reported whether they were currently in a romantic relationship and the sexual exclusivity of this relationship at baseline (0 = *not in exclusive relationship* versus 1 = *exclusive monogamous relationship*). Also at baseline, participants self-reported if they had ever tested positive for HIV and whether they had ever heard of the biomedical intervention PrEP. Sexual history was measured using a 90-day recall with a question prompting the type of sexual relationships. Participants reported whether they engaged in condomless sex regularly (i.e., never used a condom while engaging in vaginal or anal sex in the past 90 days), the number of sexual partners during the past 90 days (0 = *1 or no partners* versus 1 = *multiple partners*), and whether they exchanged sex during the past 3 months (i.e., “traded sex for money, drugs, a place to stay, food or meals, or anything else”), using items adapted from the Homeless Youth Risk and Resiliency Survey [22].

During the weeklong smartphone measurement period, participants completed a daily survey, which included a question querying the number of partners that they had sex with during the previous day (“How many people did you have vaginal or anal sex with yesterday?”) and whether they did not use a condom at any time with that partner. Furthermore, participants indicated whether they or their partner had consumed any intoxicating substance (drugs or alcohol) prior to sexual intercourse on that day. Participants provided a unique identifier (i.e., nickname) for each sexual partner on every daily survey. These unique identifiers were matched across days to determine if participants had multiple sex partners throughout the week. Participants were also asked to report if they exchanged sex for money, drugs, housing, food, or something else that day (“At any point yesterday, did you trade any type of sex (oral, vaginal, or anal)?”).

### Data Analysis

The incidence of sex events during the 7-day measurement period in the sample was observed to be relatively infrequent, with 128 days with any sexual behavior. As a result, daily logs were summarized into person-level dichotomous variables indicating whether an individual had any sex or sexual risk taking during the preceding week. The four main outcome variables were any sex (0 = *no sex* versus 1 = *any vaginal or anal sex*), condomless sex (0 = *used a condom during vaginal or anal sex at least once* versus 1 = *never used a condom*), sex with multiple partners (0 = *1 or no partners* versus 1 = *more than one partner*), and exchange sex (0 = *did not engage in exchange sex* versus 1 = *engaged in exchange sex*). Multiple logistic regressions were used to predict the likelihood of any sex (Model 1), exchange sex (Model 2), condomless sex (Model 3), and sex with multiple partners (Model 4), independently. Variables in each model were selected based on bivariate significance to the outcome or informed by existing research findings. Each logistic regression controlled for mean-centered age, gender, sexual identity, race, ethnicity, lifetime homelessness, whether the individual was currently homeless (coded as 1) or residing in a housing program (coded as 0), and relationship type at baseline (i.e., exclusive monogamous versus other). Reporting any sexual intercourse under the influence of drugs or alcohol during the week was controlled for in all models except for Model 1 due to its correlation with having any sex during the week. Positive HIV status was controlled for in all models except for Model 3 because all participants who had tested positive for HIV (*n* = 11) had sex with a condom at least once during the study period. In each model, relevant past-90-day sexual behavior was also considered as a control (e.g., in a model to predict exchange sex during the study period, we controlled for past-90-day exchange sex). Of participants recruited in the study, 20 (8.6%) completed fewer than four logs and were excluded from the analysis, leaving a final sample size of 210.

## Results

### Descriptive Statistics

As noted in Table 1, participants in the analytic sample were 22 years old on average (range = 18–27 years old), with about half identifying as male only and half identifying as heterosexual. Approximately 40% of the sample identified as Black or African American and about 40% of participants reported identifying as Hispanic. Almost 60% of the sample had at least 1 year of homelessness experience in their lifetime. One third of participants were in exclusively monogamous relationships at baseline. During the past 3 months, 75% of participants reported any sexual behavior, 9% reported engaging in exchange sex, 26% engaged in sex with no condom use, and 32% had sex with multiple partners. A positive HIV test was reported by 5.2% (*n* = 11) of the sample. Approximately 82% (*n* = 172) of participants had limited to no knowledge of PrEP. Among those who were housed (not shown in Table 1), 18 participants (nearly 17%) moved into housing in the 90-day period prior to baseline.

**Table 1:** Descriptive statistics of a sample of young adults who have experienced homelessness

	Formerly Homeless	Currently Homeless	Total	*p*
	(*n* = 109)	(*n* = 101)	(*n* = 210)	
	*n* (%)	*n* (%)	*n* (%)	
Age*	22.6 (2.5)	21.7 (2.1)	22.2 (2.3)	**.006**
Gender				**.035**
Female or other gender	59 (54.1)	40 (39.6)	99 (47.1)	
Male only	50 (45.9)	61 (60.4)	111 (52.9)	
Sexual identity				.58
Other sexual identity	53 (48.6)	53 (52.5)	106 (50.5)	
Heterosexual	56 (51.4)	48 (47.5)	104 (49.5)	
Ethnicity				**.033**
Non-Hispanic	60 (55.0)	70 (69.3)	130 (61.9)	
Hispanic	49 (45.0)	31 (30.7)	80 (38.1)	
Race				**.001**
Other race	77 (70.6)	48 (47.5)	125 (59.5)	
Black	32 (29.4)	53 (52.5)	85 (40.5)	
Lifetime homelessness				.46
Less than 1 year	42 (38.5)	44 (43.6)	86 (41.0)	
1 year or more	67 (61.5)	57 (56.4)	124 (59.0)	
Substance use				.66
Clinical CAGE indictor	50 (51.0)	45 (47.9)	95 (49.5)	
Relationship type				.86
Not in exclusive relationship	71 (65.1)	67 (66.3)	138 (65.7)	
Exclusive monogamous relationship	38 (34.9)	34 (33.7)	72 (34.3)	
Positive HIV status				.86
No	103 (94.5)	96 (95.0)	199 (94.8)	
Yes	6 (5.0)	6 (5.5)	12 (5.2)	
PREP knowledge				.61
Have never heard of it	51 (46.8)	38 (37.6)	89 (42.4)	
Have heard of it but don’t know what it is	10 (9.2)	9 (8.9)	19 (9.1)	
Know a little about it	30 (27.5)	33 (32.7)	63 (30.0)	
Know a lot about it	18 (16.5)	20 (19.8)	38 (18.1)	
Any sex (90 days)				.43
No	25 (22.9)	28 (27.7)	53 (25.2)	
Yes	84 (77.1)	73 (72.3)	157 (74.8)	
Exchange sex (90 days)				.41
No	98 (89.9)	94 (93.1)	192 (91.4)	
Yes	11 (10.1)	7 (6.9)	18 (8.6)	
Condomless sex (90 days)				.16
At least sometimes uses a condom	76 (69.7)	79 (78.2)	155 (73.8)	
Never uses a condom	33 (30.3)	22 (21.8)	55 (26.2)	
Multiple partners (90 days)				.60
1 or no partners	76 (69.7%)	67 (66.3%)	143 (68.1%)	
Multiple partners	33 (30.3%)	34 (33.7%)	67 (31.9%)	
Any sex (7 days)				.98
No	80 (73.4%)	74 (73.3%)	154 (73.3%)	
Yes	29 (26.6%)	27 (26.7%)	56 (26.7%)	
Exchange sex (7 days)				**.025**
No	105 (96.3%)	89 (88.1%)	194 (92.4%)	
Yes	4 (3.7%)	12 (11.9%)	16 (7.6%)	
Items exchanged for sex (7 days)				**.026**
Food or meals	2 (20.0%)	2 (7.7%)	4 (11.1%)	
Money	0 (0.0%)	10 (38.5%)	10 (27.8%)	
Drugs	0 (0.0%)	4 (15.4%)	4 (11.1%)	
A place to stay	0 (0.0%)	2 (7.7%)	2 (5.6%)	
Something else	8 (80.0%)	8 (30.8%)	16 (44.4%)	
Condomless sex (7 days)				.41
At least sometimes uses a condom	87 (79.8%)	85 (84.2%)	172 (81.9%)	
Never uses a condom	22 (20.2%)	16 (15.8%)	38 (18.1%)	
Multiple partners (7 days)				.19
1 or no partners	105 (96.3%)	93 (92.1%)	198 (94.3%)	
Multiple partners	4 (3.7%)	8 (7.9%)	12 (5.7%)	
Any sex under the influence (7 days)				.15
No	103 (94.5%)	90 (89.1%)	193 (91.9%)	
Yes	6 (5.5%)	11 (10.9%)	17 (8.10%)	
Average daily log compliance*	0.945 (0.10)	0.939 (0.11)	0.942 (0.10)	.66

*M (SD)

Bold values indicate the *p* < .05

Average daily compliance with diaries was high for both currently homeless and housed participants, with a mean of 94%. In the past week, about 30% of the sample reported having any sex on at least 1 day and 8% reported at least one occurrence of exchange sex. Overall, participants reported 128 days of any sexual activity of the 1410 days sampled, along with 36 events of exchange sex. The most common reason participants exchanged sex was for “something else” (44% of exchange sex events) followed by money (38% of exchange sex events). Approximately 18% of participants never utilized a condom during any sex event during the measurement week, 8% had at least one sexual event under the influence during the week, and 6% had sex with more than one partner during the week. There were significant differences between those in housing programs compared to currently homeless participants, as seen in Table 1. Compared to those in housing, participants who were experiencing homelessness were approximately 1 year younger and were more likely to identify as male only, less likely to identify as Hispanic, and more likely to identify as Black. Currently homeless participants were more likely to report engaging in exchange sex during the study week, and the items exchanged were significantly different among participants who were currently homeless, including money (39% of exchange sex events), drugs (15% of exchange sex events), a place to stay (8%), or food (8%). Participants who were currently housed who engaged in exchange sex did so for “something else” or food exclusively. There were no significant differences in other past-90-day sexual history or other sexual behaviors during the week between currently homeless participants and participants in housing programs.

### Primary Outcomes

Results from models predicting any sex (Model 1), exchange sex (Model 2), condomless sex (Model 3), and sex with multiple partners (Model 4) during the 7-day measurement period are reported in Table 2. Compared to those reporting no sex in the past 90 days, those who reported having any sex in the past 90 days experienced a 5.48-point increase in the odds (95% CI = 1.51, 19.88) of reporting any sex on at least 1 day of the measurement period after adjusting for all covariates (*p* = 0.01). Participants in exclusively monogamous relationships had higher odds (*OR* = 3.57; 95% CI = 1.69, 7.57) of reporting having sex on at least 1 day during the measurement period than participants not in an exclusive relationship (*p* = 0.001). Male participants compared to other gender identities, however, experienced a decrease in the odds of reporting any sex during the weeklong measurement period (*OR* = 0.40; 95% CI = 0.18, 0.87; *p* = 0.02). There were marginally significant decreased odds of reporting having any sex during the measurement period with each additional year of age (*p* = 0.10) and for those identifying as heterosexual (*p* = 0.092) and Hispanic (*p* = 0.068).

**Table 2:** Results of logistic regressions predicting sex events occurring at least once in a 7-day measurement period in a sample of young adults who have experienced homelessness (*N* = 210)

	Model 1: Any Sex	Model 2: Exchange Sex	Model 3: Condomless Sex	Model 4: Multiple Partners
	(*n* = 56)	(*n* = 16)	(*n* = 38)	(*n* = 12)
	*OR* (95% CI)	*OR* (95% CI)	*OR* (95% CI)	*OR* (95% CI)
Age (centered)	0.87 (0.74, 1.03)*	1.07 (0.82, 1.39)	0.93 (0.77, 1.12)	0.94 (0.68, 1.31)
Gender				
Male only	0.40 (0.18, 0.87)**	0.86 (0.28, 2.65)	0.84 (0.34, 2.13)	2.02 (0.45, 9.03)
Sexual identity				
Heterosexual	1.93 (0.90, 4.13)*	0.49 (0.16, 1.48)	1.01 (0.42, 2.43)	5.04 (0.95, 26.88)**
Ethnicity				
Hispanic	2.12 (0.95, 4.74)*	2.65 (0.79, 8.86)	1.28 (0.49, 3.13)	1.94 (0.49, 7.65)
Race				
Black	0.84 (0.37, 1.90)	3.04 (0.85, 10.82)*	1.42 (0.54, 3.77)	0.70 (0.17, 2.98)
Lifetime homelessness				
1 year or more	1.82 (0.81, 4.07)	4.03 (1.10, 14.79)**	1.25 (0.50, 3.12)	1.73 (0.34, 8.69)
Housing status				
Currently homeless	1.26 (0.60, 2.66)	3.23 (0.98, 10.65)**	0.57 (0.23, 1.40)	1.97 (0.48, 8.10)
Relationship type				
Exclusive monogamous relationship	3.57 (1.69, 7.57)***	0.77 (0.23, 2.57)	3.30 (1.32, 8.24)***	0.40 (0.06, 2.52)
Positive HIV status				
Yes	0.57 (0.10, 3.28)	10.55 (1.77, 63.08)**	–	0.85 (0.06, 11.62)
Any sex (90 days)				
Yes	5.48 (1.51, 19.88)**	–	–	–
Exchange sex (90 days)				
Yes	–	3.56 (0.75, 16.87)	–	–
Condomless sex (90 days)				
Never uses a condom	–	–	2.90 (1.16, 7.22)**	–
Multiple partners (90 days)				
Multiple partners	–	–	–	3.13 (0.81, 12.12)*
Sex under the influence (7 days)	–	1.53 (0.29, 8.09)	14.24 (3.81, 53.25)***	3.25 (0.49, 21.44)

*p < .10.; **p < .05.; ***p < .01

Participants who identify as Black were marginally more likely to report exchange sex on at least 1 day of the measurement period than those identifying as other racial identities (*p* = 0.086). Those who had spent a year or more unstably housed during their lifetime experienced a 4.03-point increase (95% CI = 1.10, 14.79) in the odds of having exchange sex during the week than participants who reported less than a year of lifetime homelessness experience (*p* = 0.036). In addition, participants who tested positive for HIV experienced more than a 10-point increase in the odds (95% CI = 1.77, 63.08) of reporting exchange sex during the week than those who did not report a confirmed positive test of HIV (*p* = 0.010). After adjusting for all covariates, participants currently experiencing homelessness had 3.23 times (95% CI = 0.98, 10.65) higher odds of reporting exchange sex on at least 1 day of the measurement period than those who were formerly homeless and now residing in housing programs (*p* = 0.054).

Participants who did not use a condom during any reported sex events during the 90 days prior to baseline experienced a 2.90-point increase in the odds (95% CI = 1.16, 7.22) of never using a condom during sexual intercourse throughout the measurement week (*p* = 0.022). Participants in exclusively monogamous relationships had, on average, a 3.30-point increase in the odds (95% CI = 1.32, 8.24) of reporting condomless sex during the measurement period than participants not in an exclusive relationship (*p* = 0.010). Furthermore, participants who had sexual intercourse under the influence at least once during the week experienced much higher odds (*OR* = 14.24; 95% CI = 3.81, 53.25) in reporting condomless sex during the study week (*p* < 0.001).

Last, in Model 4, participants who identify as heterosexual experienced a 5.04-point increase in the odds (95% CI = 0.95, 26.88), trending toward significance, of reporting sex with multiple partners during the measurement period than participants identifying as other sexual identities (*p* = 0.058). There were marginally significant increased odds of reporting having sex with multiple partners during the measurement period if participants had sex with multiple partners in the 90 days prior to baseline (*p* = 0.098).

## Discussion

This study describes predictors of risky sexual behavior among YAEH—who were either currently or formerly homeless and in supportive housing—through the use of mobile phone-based daily assessment methods. Momentary and daily assessment through EMA has been increasingly utilized to understand exposures and outcomes in HIV research [45], because use of real-time measures is known to improve validity though reducing recall bias, limit social desirability bias [46], and provide more complete data of discrete behaviors [47]. Our findings provide some guidance on the advantages and limitations to daily assessment methods of sexual activity in this population; namely, although daily assessments are feasible and can provide contextual indicators of sexual risk, a 7-day measurement period does not necessarily represent an adequate timeframe to capture risky sexual behavior in this population. Of 1410 study days across our sample, sexual activity was relatively infrequent, which precluded the use of other momentary data. This suggests two possible lessons. The first is that that studies of YAEH using intensive longitudinal designs should recruit only participants who frequently engage in risky behaviors. Though the majority of our sample (75%) reported sexual activity in the 90 days leading up the 7-day measurement period, only 30% of participants self-reported sexual activity in daily assessments. In the 90 days prior to the study, 26% of the sample engaged in sexual activity in which they never utilized a condom and 32% had sexual intercourse with multiple partners, as opposed to 18% and 6%, respectively, during the study week. The second possible lesson is that studies of YAEH using intensive longitudinal designs should be designed to follow participants for longer than 1 week, which requires balancing the collection of relevant contextual information with respondent burden [48]. In the only other known study of currently homeless young adults that used a similar daily survey approach, participants were followed for 3 weeks, with 70% reporting being sexually active and approximately 4 of 5 engaging in high-risk sexual activities, including having condomless sex, having multiple sexual partners on the same day, and engaging in exchange sex [49].

For the sex that did occur during our study’s 1-week measurement period, predictors included recent sexual intercourse during the past 90 days and monogamy. Similar to the general population, attachment to a monogamous partner is indicative of more frequent sexual behavior [40]. For the two risky sexual behaviors of condomless sex and sex with multiple partners, predictors included monogamy, past risky behavior, and sexual identity. Alarmingly, participants who had sexual intercourse under the influence of drugs or alcohol also had a higher odds of reporting condomless sex throughout the week. There were no differences based on participant housing status during either the previous 90 days or during the measurement week in both condomless sex rates or sex with multiple partners, nor did we find a difference in PREP knowledge or HIV prevalence [3]. Though we found a slightly lower self-reported HIV rate of 5% compared to a measured seroprevalence of ~ 10% [3, 50], HIV preventive services appear to be needed in both housing programs and among other homeless service providers because close to one third of participants still engaged in risky sexual behavior, including routinely having sex without a condom and sex with multiple partners, and the fact that we found increased odds of reporting exchange sex during the week if a participant had a positive diagnosis of HIV.

In line with past research, approximately 10% of our sample reported exchanging sex either in the 90 days leading up to the study or during the assessment period [51]. Rates of exchange sex did not differ based on housing status during the 90 days prior to baseline; however, young adults who were currently experiencing homelessness were significantly more likely to report exchange sex in daily diaries compared with those who resided in supportive housing programs. This likely points to the role exchange sex plays in survival among those who are actively homeless, often a direct result of lack of resources and exclusion from mainstream economic opportunities. In fact, every additional year of homelessness increases this population’s likelihood of engaging in survival sex by 10% [19]. Thus, it is fitting that our findings indicate that youth who had spent more than a year unstably housed during their lifetime were significantly more likely to report exchange sex during the study week. These findings indicate that housing status may protect against exchange sex, or at least reduce the frequency and reasons for why participants engage in exchange sex. During the measurement period, currently homeless participants reported exchanging sex for money and a place to stay—neither of which were endorsed by housed participants who had exchanged sex. Though overall rates of exchange sex did not change between 90-day recall and the measurement week, housing status affected the odds of reporting current exchange sex behavior. It is also possible that during the 90-day period prior to baseline, the 18 participants who received housing during that period no longer found the need to exchange sex to meet basic needs, such as shelter. After being placed in housing programs, individuals typically experience economic stability, access to services, community, and overall improvement in quality of life.

This study was primarily limited by a reliance on a convenience sample of young adults recruited from only one U.S. metropolitan area. Prior sexual behavior and thus, risk of HIV was not viewed as an eligibility requirement for this study. Approximately one quarter of the sample did not report any sexual activity during the 90 days leading up to the study. Future studies may seek to include larger samples, longer measurement periods, and only individuals engaged in behavior that is associated with risk or HIV or sexually transmitted infections, such as individuals who regularly engage in unprotected sexual activity with multiple partners or do not regularly utilize condoms. The study confounded between- and within-subject effects as a result of infrequent sexual activity during the measurement period. Significant and marginally significant predictors should be viewed in light of the infrequency of captured sexual risk behaviors, as evidenced by large confidence intervals in our findings. The inclusion of individuals with a greater incidence of high-risk behavior and a larger sample size may facilitate within-subject findings and allow for inference of sexual risk taking based on momentary factors, such as substance use, stress, or emotional affect [52]. Future research focused on relatively rare events such as sex may benefit from employing event-based reporting in which individuals only respond to assessments when engaged in a behavior [52, 53]. In addition, longer measurement periods may be warranted. Employing a 7-day measurement period is common in other EMA studies of health risk behaviors but may be insufficient to study sexual risk taking. This may explain why frequency of sexual risk taking was lower in daily diaries than in the 90 days prior to baseline, even though our participants displayed high compliance with daily assessments. Future studies will need to weigh the benefit of a longer measurement period with increased participant burden; however, burden can be reduced by the use of event-based reporting and short assessments. Pilot work may also address the ideal measurement timeframe to address sexual risk taking because a week seems to be insufficient. Regarding exchange sex, future research should better address daily factors that may foster or protect against this behavior, such as hunger, social environment, or lack of any shelter, collected via daily recall methods and ideally, mobile-based methods that can collect detailed information about the geographical context. In our survey design, we listed four options we hypothesized a young adult may select as having exchanged sex (i.e., food, money, drugs, or a place to stay). Nonetheless, participants most commonly endorsed “something else” (i.e., an item not listed). More research is needed to determine the basic needs youth are meeting by exchanging sex, particularly after moving into supportive housing. Moreover, because housing protects against exchange sex, longitudinal studies are needed to follow young adults as their living environment changes from the street to supportive housing.

Although findings indicate that general sexual activity may not be more frequent among young adults who have experienced homelessness than those in the general population, exchange sex is still prevalent in this population, which increases the likelihood of contracting HIV. Access to stable housing has been shown to improve functioning. Not only is stable housing associated with increased positive outcomes at discharge, but engagement in the services offered via supportive housing has been proven to increase outcomes across domains, including educational attainment, employment, life skills, and quality of life [54–57]. Given this, the stability offered by housing may also foster healthier sexual behaviors. Even so, YAEH face extreme poverty and barriers to meeting their basic needs, increasing their likelihood of exchange sex and its associated risks [24]. To combat these risks, this study provides additional evidence that it is essential we continue to focus on supportive housing as a primary intervention for homelessness, because access to a safe and stable place to live with built-in supports has potential to not only provide basic housing needs but also support behavioral health through minimizing risk. In the future, HIV prevention efforts outside of housing may focus on job skills programs in drop-in centers to improve economic opportunities for YAEH to minimize participation in the street economy, such as exchanging sex. Programming for YAEH may benefit from an increased focus on the economic link between homelessness and exchange sex. Increasing access to basic needs such as housing, food, and hygiene items may be a simple but highly effective way to support YAEH in meeting their daily needs, rather than needing to trade sex. Additional harm reduction approaches to substance use in YAEH practice and policy can also reduce HIV risk and survival and exchange sex by providing another, safe means for obtaining sought-after materials—a known approach to reducing HIV infection [58]. To further support these efforts in practice, there is a continued need for psychoeducation regarding harm reduction, healthy sexual relationships, and safer sex behaviors, including routine condom use even in the context of monogamous relationships, the link between sexual risk taking and substance use, and specialized interventions to limit exchange sex [24].

## References

[1] Morton MH, Dworsky A, Matjasko JL, Curry SR, Schlueter D, Chávez R (2018). Prevalence and correlates of youth homelessness in the United States. J Adolesc Health.

[2] Gamarel KE, Brown L, Kahler CW, Fernandez MI, Bruce D, Nichols S (2016). Prevalence and correlates of substance use among youth living with HIV in clinical settings. Drug Alcohol Depend.

[3] Pfeifer RW, Oliver J (1997). A study of HIV seroprevalence in a group of homeless youth in Hollywood, California. J Adolesc Health.

[4] Kipke MD, Montgomery SB, Simon TR, Unger JB, Johnson CJ (1997). Homeless youth: drug use patterns and HIV risk profiles according to peer group affiliation. AIDS Behav.

[5] Rotheram-Borus MJ, Song J, Gwadz M, Lee M, Van Rossem R, Koopman C (2003). Reductions in HIV risk among runaway youth. Prev Sci.

[6] Lucenko BA, Sharkova IV, Huber A, Jemelka R, Mancuso D (2015). Childhood adversity and behavioral health outcomes for youth: an investigation using state administrative data. Child Abuse Negl.

[7] Abrams LS, Curry SR, Lalayants M, Montero L (2017). The influence of policy context on transition age foster youths’ views of self-sufficiency. J Soc Serv Res.

[8] Crawford BL, McDaniel J, Moxley D, Salehezadeh Z, Cahill AW (2015). Factors influencing risk of homelessness among youth in transition from foster care in Oklahoma: implications for reforming independent living services and opportunities. Child Welfare.

[9] Curry SR, Abrams LS (2015). Housing and social support for youth aging out of foster care: state of the research literature and directions for future inquiry. Child Adolesc Soc Work J.

[10] Fowler PJ, Toro PA, Miles BW (2009). Pathways to and from homelessness and associated psychosocial outcomes among adolescents leaving the foster care system. Am J Public Health.

[11] Shah MF, Liu Q, Mark Eddy J, Barkan S, Marshall D, Mancuso D (2017). Predicting homelessness among emerging adults aging out of foster care. Am J Community Psychol.

[12] Omura JD, Wood E, Nguyen P, Kerr T, DeBeck K (2014). Incarceration among street-involved youth in a Canadian study: implications for health and policy interventions. Int J Drug Policy.

[13] Petering R, Rice E, Rhoades H (2016). Violence in the social networks of homeless youths: implications for network-based prevention programming. J Adolesc Res.

[14] Petering R, Rice E, Rhoades H, Winetrobe H (2014). The social networks of homeless youth experiencing intimate partner violence. J Interpers Violence.

[15] Wenzel SL, Koegel P, Gelberg L (2000). Antecedents of physical and sexual victimization among homeless women: a comparison to homeless men. Am J Community Psychol.

[16] Narendorf SC, Cross MB, Santa Maria D, Swank PR, Bordnick PS (2017). Relations between mental health diagnoses, mental health treatment, and substance use in homeless youth. Drug Alcohol Depend.

[17] Nyamathi A, Hudson A, Greengold B, Slagle A, Marfisee M, Khalilifard F (2010). Correlates of substance use severity among homeless youth. J Child Adolesc Psychiatr Nurs.

[18] Thompson SJ, Bender K, Ferguson KM, Kim Y (2015). Factors associated with substance use disorders among traumatized homeless youth. J Soc Work Pract Addict.

[19] Walls NE, Bell S (2011). Correlates of engaging in survival sex among homeless youth and young adults. J Sex Res.

[20] Heerde JA, Hemphill SA (2016). Sexual risk behaviors, sexual offenses, and sexual victimization among homeless youth: a systematic review of associations with substance use. Trauma Violence Abuse.

[21] Flom PL, Friedman SR, Kottiri BJ, Neaigus A, Curtis R, Des Jarlais DC (2001). Stigmatized drug use, sexual partner concurrency, and other sex risk network and behavior characteristics of 18- to 24-year-old youth in a high-risk neighborhood. Sex Transm Dis.

[22] Maria DMS, Flash C, Narendorf S, Drake S, Barman-Adhikari A, Petering R (2018). Knowledge and attitudes about prep and Npep among a 7-city sample of homeless young adults. J Adolesc Health.

[23] Heerde JA, Hemphill SA (2017). The role of risk and protective factors in the modification of risk for sexual victimization, sexual risk behaviors, and survival sex among homeless youth: a meta-analysis. J Investig Psychol Offender Profiling.

[24] Murphy LT (2016). Labor and sex trafficking among homeless youth.

[25] Wenzel SL, Rhoades H, La Motte-Kerr W, Duan L, Harris T, Rice E (2019). Do HIV risk and prevention behaviors change over time among adults in permanent supportive housing?. AIDS Care.

[26] Wolitski RJ, Kidder DP, Pals SL, Royal S, Aidala A, Stall R (2010). Randomized trial of the effects of housing assistance on the health and risk behaviors of homeless and unstably housed people living with HIV. AIDS Behav.

[27] Curry SR, Abrams LS (2015). “They lay down the foundation and then they leave room for us to build the house”: a visual qualitative exploration of young adults’ experiences of transitional housing. J Soc Social Work Res.

[28] Henwood BF, Redline B, Semborski S, Rhoades H, Rice E, Wenzel SL (2018). What’s next? A theory on identity preservation for young adults in supportive housing. Cityscape.

[29] Holtschneider C (2016). A part of something: the importance of transitional living programs within a Housing First framework for youth experiencing homelessness. Child Youth Serv Rev.

[30] Kisely SR, Parker JK, Campbell LA, Karabanow J, Hughes JM, Gahagan J (2008). Health impacts of supportive housing for homeless youth: a pilot study. Public Health.

[31] Kozloff N, Adair CE, Lazgare LIP, Poremski D, Cheung AH, Sandu R (2016). “Housing first” for homeless youth with mental illness. Pediatrics..

[32] Dodd SJ, Ruffins J, Arzola D (2018). Improving health while saving money: lessons learned from a supportive housing program for young adults with HIV. Sex Res Soc Policy.

[33] National Academies of Sciences E. Permanent supportive housing: evaluating the evidence for improving health outcomes among people experiencing chronic homelessness [Internet]. 2018. Available from: https://www.nap.edu/catalog/25133/permanent-supportive-housing-evaluating-the-evidence-for-improving-health-outcomes. Accessed on 14 Nov 2019 30148580

[34] Smyth JM, Stone AA (2003). Ecological momentary assessment research in behavioral medicine. J Happiness Stud.

[35] Shiffman S (2014). Conceptualizing analyses of ecological momentary assessment data. Nicotine Tob Res.

[36] Rice E, Lee A, Taitt S (2011). Cell phone use among homeless youth: potential for new health interventions and research. J Urban Health.

[37] Graham CA, Catania JA, Brand R, Duong T, Canchola JA (2003). Recalling sexual behavior: a methodological analysis of memory recall bias via interview using the diary as the gold standard. J Sex Res.

[38] Napper LE, Fisher DG, Reynolds GL, Johnson ME (2010). HIV risk behavior self-report reliability at different recall periods. AIDS Behav.

[39] Coxon APM (1999). Parallel accounts? Discrepancies between self-report (diary) and recall (questionnaire) measures of the same sexual behaviour. AIDS Care.

[40] McAuliffe TL, DiFranceisco W, Reed BR (2007). Effects of question format and collection mode on the accuracy of retrospective surveys of health risk behavior: a comparison with daily sexual activity diaries. Health Psychol.

[41] U.S. Government Publishing Office. McKinney-Vento Homeless Assistance Act of 1986. Washington, DC; 1986. (101 Stat. 482). Report No.: Pub, L. 100-77.

[42] Henwood BF, Redline B, Dzubur E, Madden DR, Rhoades H, Dunton GF (2019). Investigating health risk environments in housing programs for young adults: protocol for a geographically explicit ecological momentary assessment study. JMIR Res Protoc.

[43] Sexual Assessment Research Team (SMART) (2009). Best practices for asking questions about sexual orientation on surveys.

[44] Ewing JA (1984). Detecting alcoholism. The CAGE questionnaire. JAMA.

[45] Smiley SL, Milburn NG, Nyhan K, Taggart T (2020). A systematic review of recent methodological approaches for using ecological momentary assessment to examine outcomes in U.S. based HIV research. Curr HIV/AIDS Rep.

[46] Latkin CA, Mai NVT, Ha TV, Sripaipan T, Zelaya C, Le Minh N (2016). Social desirability response bias and other factors that may influence self-reports of substance use and HIV risk behaviors: a qualitative study of drug users in Vietnam. AIDS Educ Prev.

[47] Rowe C, Hern J, DeMartini A, Jennings D, Sommers M, Walker J (2016). Concordance of text message ecological momentary assessment and retrospective survey data among substance-using men who have sex with men: a secondary analysis of a randomized controlled trial. JMIR Mhealth Uhealth.

[48] Stone A, Shiffman S, Atienza A, Nebeling L (2007). The science of real-time data capture: self-reports in health research.

[49] Maria DS, Padhye N, Yang Y, Gallardo K, Businelle M (2018). Predicting sexual behaviors among homeless young adults: ecological momentary assessment study. JMIR Public Health Surveill.

[50] Yadav A, Soriano Marcolino L, Rice E, Petering R, Winetrobe H, Rhoades H, et al. Preventing HIV spread in homeless populations using PSINET:emerging application case study. In: Proceedings of the 27th Conference on Innovative Applications of Artificial Intelligence (IAAI 2015) [Internet]. IAAI; 2015. p. 4006–11. Available from https://eprints.lancs.ac.uk/id/eprint/81436/. Accessed on 10 Jun 2020

[51] Halcón LL, Lifson AR (2004). Prevalence and predictors of sexual risks among homeless youth. J Youth Adolesc.

[52] Blood EA, Shrier LA (2013). The temporal relationship between momentary affective states and condom use in depressed adolescents. Arch Sex Behav.

[53] Swendeman D, Comulada WS, Ramanathan N, Lazar M, Estrin D (2015). Reliability and validity of daily self-monitoring by smartphone application for health-related quality-of-life, antiretroviral adherence, substance use, and sexual behaviors among people living with HIV. AIDS Behav.

[54] Gwadz MV, Cleland CM, Leonard NR, Bolas J, Ritchie AS, Tabac L (2017). Understanding organizations for runaway and homeless youth: a multi-setting quantitative study of their characteristics and effects. Children Youth Services Rev.

[55] Pierce SC, Grady B, Holtzen H (2018). Daybreak in Dayton: assessing characteristics and outcomes of previously homeless youth living in transitional housing. Child Youth Serv Rev.

[56] Raithel J, Yates M, Dworsky A, Schretzman M, Welshimer W (2015). Partnering to leverage multiple data sources: preliminary findings from a supportive housing impact study. Child Welfare.

[57] Rashid S (2004). Evaluating a transitional living program for homeless, former foster care youth. Res Soc Work Pract.

[58] Van Den Berg C, Smit C, Van Brussel G, Coutinho R, Prins M (2007). Amsterdam Cohort. Full participation in harm reduction programmes is associated with decreased risk for human immunodeficiency virus and hepatitis C virus: evidence from the Amsterdam Cohort Studies among drug users. Addiction.

